# School closures and well-being-related topic searches on Google during the COVID-19 pandemic in Sub-Saharan Africa

**DOI:** 10.1186/s12889-023-16186-6

**Published:** 2023-06-27

**Authors:** Uchechi Shirley Anaduaka, Ayomide Oluwaseyi Oladosu

**Affiliations:** 1 Department of Accountancy, Economics and Finance, Hong Kong Baptist University, 34 Renfrew Road, Kowloon Tong, Hong Kong; 2 School of Graduate Studies, Lingnan University, 8 Castle Peak Road, Tuen Mun, Hong Kong Hong Kong

**Keywords:** COVID-19, School Closures, Well-being, Google Trends, Empirical methods, Sub-Saharan Africa

## Abstract

**Background:**

Following the outbreak of the 2020 coronavirus, governments adopted non-pharmaceutical interventions (NPIs) to save lives. The NPIs have been deemed to have unintended consequences on mental health and well-being. This study aimed to estimate the impact of the COVID-19 pandemic-induced school closures on the relative search volumes (RSVs) of well-being-relevant topics in 30 low and lower- middle income countries in Sub-Saharan Africa.

**Methods:**

Google Trends search data, difference-in-differences and event study methods were used to evaluate the impact on the related search volume (RSV) of well-being related topic queries in Sub-Saharan Africa.

**Results:**

The results suggest positive and significant increases in the search intensity for anger, boredom, fear, sleep, exercise, and prayer. Contrary to other studies, we find no discernible effects on the relative search volumes (RSVs) on loneliness, sadness, and suicide.

**Conclusion:**

Our findings suggest that the pandemic and the associated restrictions had a mixed effect on well-being-related searches. We recommend increased vigilance and proactive communication from the government and policy makers with the general population in times of emergencies when social policies that restrict lives and liberties need to be adopted.

**Supplementary Information:**

The online version contains supplementary material available at 10.1186/s12889-023-16186-6.

## Introduction

The coronavirus disease (COVID-19) impacted people across the globe and dramatically changed lives, impacted livelihoods and restricted liberties at an unprecedented speed and scale [1]. Given the pathogen’s exponential growth and the absence of a vaccine, governments in sub-Saharan Africa (SSA) implemented strict non-pharmaceutical interventions (NPIs), e.g. lockdowns with all but essential internal travel banned, mandated or voluntary work-from-home, closures of school, non-essential businesses and international borders, bans on large gatherings in religious institutions, entertainment and sport centres, curfews and quarantines to isolate symptomatic individuals and their contacts, as well as behavioural changes (e.g., the use of face masks in public) [2–4]. This was done in a bid to flatten the curve and reduce patients’ burden on the largely underfunded and already strained health system as they dealt with the pandemic’s effects [5].

Studies have indicated that the restriction of liberty, morbidity and loss of lives due to COVID-19 have far-reaching implications on the mental health and subjective well-being of the populace [6–8]. For instance, the pandemic and the resultant measures affected people’s employment status, created financial insecurity, isolated individuals from their social circles, leading to loneliness, and exacerbated pre-existing mental problems due to the fear of contracting the virus, anxiety about the future, and grief from losing loved ones [9–12]. The state of one’s mental health and subjective well-being could affect their choices, behaviours and outcomes, thereby potentially impacting their future physical health and longevity [13].

This paper estimates the impact of the COVID-19 pandemic-induced school closures on the relative search volumes (RSVs) of well-being-relevant topics in 30 low- and lower- middle-income countries in SSA. The study adopts graphical analyses, difference-in- differences (DiD) and event study to assess the association between the timing of school closures and changes in topic searches. The data was sourced from Google Trends® (GT), a publicly available real-time source of data that can be useful in analysing public reactions to social policies when traditional (questionnaires and face- to-face interviews) or virtual (phone surveys, web surveys and video conferencing) methods cannot be frequently used, are costly, time-consuming, and limited in scope, e.g., in emergencies where participants are hard-to-reach or hard-to-survey [14]. This study, to our knowledge, is the first to estimate the impact of the COVID- 19 pandemic on well-being-related GT searches in SSA. The study contributes to the literature on the impact of COVID-19 on well-being, particularly on those that use GT data to answer research questions on health and social issues [7, 8, 11, 15].

The rest of the paper is structured as follows. Section 2 describes the study’s context and the literature. Section 3 describes the data and the analytical strategy. Section 4 presents the results, Sect. 5 discusses the study’s findings, strengths and limitations. Finally, Sect. 6 concludes the study.

### Context of the study

#### COVID-19 pandemic and government responses in Sub-Saharan Africa

The 2020 coronavirus outbreak, declared a pandemic on March 11 2020, is the largest challenge to public health in this century [16]. As of December 2021, over 280 million people had been infected, and over 5 million deaths reported globally [17]. In SSA, the first case was confirmed on February 28, 2020, in Nigeria [18], and the first death was reported on March 13, 2020, in Sudan [16] (Table 1 below). Following the first case in Nigeria, other SSA countries in this study confirmed their first cases between March 2 and May 13, 2020. As of March 9, 2020, the first ten cases in SSA were confirmed and reported in Cameroon, Nigeria, Senegal, South Africa and Togo. As of March 22, 2020, the first ten deaths were confirmed and were reported in Burkina Faso, the Democratic Republic of Congo, Gabon, Ghana, Mauritius, and Sudan. As of September 2020, more than one million confirmed COVID-19 cases and more than 25,000 associated deaths were reported, with South Africa, Ethiopia, and Nigeria having more than 50,000 cases and 1,000 deaths [19].

Regarding NPIs, school closures were first initiated in Mauritius on March 10, 2020 [20]. By March 31, 2020, 38 countries had closed their educational institutions disrupting learning activities for over 237 million students [20]. The length of the school closures varied among the SSA countries, with some countries introducing partial openings within one month (e.g., Madagascar) to nine months (e.g., Sudan). To ensure continuity of learning, countries introduced distance learning modalities, including instruction through radio, TV and online channels [20]. In addition to school closures, several countries had localised or national lockdowns. For instance, the Democratic Republic of Congo first initiated a localised lockdown on March 19, 2020, and Rwanda initiated a full lockdown two days later [21] (Table 2 below). Among the 30 countries in this study, only eight adopted nationalised lockdowns (Angola, Djibouti, Kenya, Lesotho, Republic of Congo, Rwanda, Uganda, and Zimbabwe) [21].

**Table 1:** Starting dates of COVID-19 in Sub-Saharan Africa and the world

	World	SSA	Nigeria	Sudan
Date of first case	17/11/19	28/02/20	28/02/20	14/03/20
First 10 cases	22/01/20a	09/03/20	20/03/20	03/04/20
Date of first death	09/01/20	13/03/20	23/03/20	13/03/20
First 10 deaths	22/01/20	22/03/20	11/04/20	18/04/20
Lockdownb	23/01/20c	19/03/20	30/03/20	30/03/20
School closured	16/02/20	10/03/20	26/03/20	16/03/20

Notes: The dates are compiled from: The Guardian, 2020 [22]; [19]; [20] & [23]. The countries are defined by the African Union (https://au.int/en/memberstates/countryprofiles2). (a) The exact date is unknown. However, by January 22 2020, more than 500 people had been confirmed in China [19]. (b) Lockdown is defined here as localised or national lockdown. (c) The Chinese government initiated a national response in the Hubei province as it banned travel to and from Wuhan and enforced strict quarantines in the affected regions [24]. (d) Mongolia was the first country to close schools globally [20]

**Table 2:** Starting dates of the COVID-19 pandemic in Sub-Saharan Africa

Sub- region	Country	Date of 1st case	Average stringency Index	Date when schools were fully closed	Date when schools were partially opened	Date when schools were fully opened	Lockdown^a^
**Panel A: Low-Income Countries**							
Western	Burkina Faso	10/03/20	41.05	16/03/20	02/06/20	01/10/20	Localised
Central	Congo, Dem. Rep	11/03/20	49.80	19/03/20	10/08/20	12/10/20	Localised
Eastern	Ethiopia	13/03/20	53.75	23/03/20	20/10/20	04/10/21	Localised
Western	The Gambia	17/03/20	50.44	18/03/20	24/06/20	28/10/20	No
Western	Guinea	13/03/20	52.46	25/03/20	n/a	01/09/20	Localised
Western	Liberia	16/03/20	53.70	16/03/20	29/06/20	30/11/20	Localised
Eastern	Madagascar	20/03/20	49.69	21/03/20	22/04/20	01/09/21	No
Southern	Malawi	02/04/20	41.51	23/03/20	14/07/20	22/02/21	No
Southern	Mozambique	22/03/20	46.20	23/03/20	01/10/20	30/08/21	No
Western	Niger	20/03/20	25.41	23/03/20	n/a	15/10/20	Localised
Eastern	Rwanda	14/03/20	57.48	16/03/20	07/10/20	02/08/21	National
Eastern	Somalia	16/03/20	32.14	18/03/20	n/a	01/04/21	No
Eastern	Sudan	14/03/20	55.64	16/03/20	22/11/20	22/02/22	No
Western	Togo	06/03/20	41.87	20/03/20	08/06/20	02/11/20	Localised
Eastern	Uganda	21/03/20	61.32	20/03/20	15/10/20	10/01/22	National
**Panel B: Lower-Middle-Income Countries**							
Southern	Angola	20/03/20	53.42	24/03/20	05/10/20	10/02/21	National
Western	Benin	16/03/20	35.92	30/03/20	11/05/20	28/09/20	Localised
Central	Cameroon	06/03/20	42.52	18/03/20	01/06/20	15/10/20	No
Central	Congo, Rep	15/03/20	52.05	19/03/20	18/05/20	12/10/20	National
Western	Cote d’Ivoire	11/03/20	42.92	17/03/20	18/05/20	14/09/20	Localised
Eastern	Djibouti	18/03/20	46.39	20/03/20	n/a	07/09/20	National
Southern	Eswatini	14/03/20	57.16	19/03/20	06/07/20	08/09/21	Localised
Western	Ghana	14/03/20	41.77	16/03/20	15/06/20	10/03/21	Localised
Eastern	Kenya	13/03/20	59.04	16/03/20	19/10/20	04/01/21	National
Southern	Lesotho	13/05/20	49.01	19/03/20	18/05/20	12/04/21	National
Western	Nigeria	28/02/20	54.06	26/03/20	21/09/20	02/11/20	Localised
Western	Senegal	02/03/20	40.49	16/03/20	25/06/20	12/11/20	Localised
Eastern	Tanzania	16/03/20	26.78	19/03/20	01/06/20	29/06/20	Localised
Southern	Zambia	18/03/20	35.07	20/03/20	02/06/20	23/08/21	No
Southern	Zimbabwe	20/03/20	55.40	24/03/20	14/09/20	06/09/21	National

*Notes*: Income classification comes from the World Bank Country and Lending Groups; *LI* are low-income countries with $1,035 or less; *LMI* are lower-middle-income countries with $1,036 to $4,045; *UMI* are upper- middle-income countries with income $4,046 to $12,535, and *HI* are high-income countries with income $12,536 or more. ^a^ This is defined as whether the country had imposed a lockdown by April 2020

#### COVID-19’s impact on well-being

Mental health and well-being are essential for individuals to realise their potential, cope with the normal stresses of life, work productively, and contribute fruitfully to their community [25]. Poor mental health and well-being adds to the global burden of disease, lowers the quality of life and have substantial socioeconomic costs [26]. Extreme events such as COVID-19 have direct and indirect implications for mental health and well-being [27, 28]. For instance, the stringent NPIs implied unprecedented loss of usual routine, changes in day-to-day lives, disruption in learning, loss of jobs and sources of income, an increase in informal employment, and physical isolation from families, friends, and acquaintances [10, 29–33]. The by-products of school closures such as unemployment, social isolation (due to disruption in learning and entertaining), and lack of freedom, are common risk factors for social dysfunction and poor well-being [12, 34, 35]. For young people, feelings of fear, anger, sadness and boredom were among the emotions shown during this period [31, 36], as the restrictions prevented them from attending school in-person and having physical interactions with peers [37]. Although positive steps can be taken to improve well-being (learning new skills, staying close to family members and friends virtually, doing exercise and expressing love to friends), they require mental fortitude to cope psychologically with the new normal stresses of life [38].

## Materials and methods

### Sample and study design

**Fig. 1:**
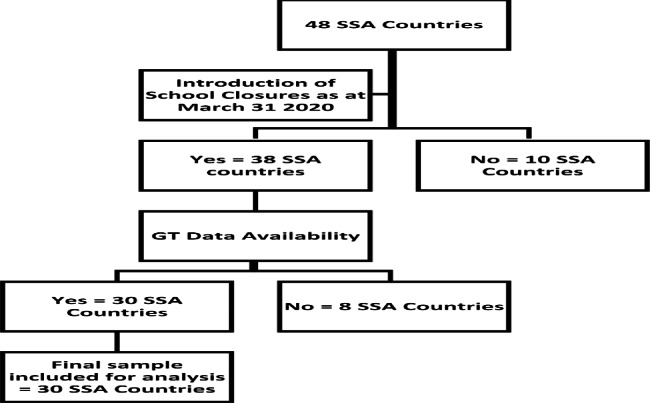
Flowchart of the sample selection process

This paper estimates the association between COVID-19 school closures and well- being-related topic searches in 30 SSA countries (Table 2 below). The 30 countries were purposively sampled from the list of 48 countries across the four regions of SSA (Central, Eastern, Southern, and Western Africa) [39], based on the availability of the Google Trends data for the topics under investigation. In addition, these countries were among the 38 countries that had imposed school closures as of March 31, 2020 [20] following the declaration of COVID-19 as a pandemic [16]. Please refer to Fig. 1 below for the flowchart depicting the sample selection process.

### Data source and data query

The data was extracted using the GTrends R package [40, 41], which automatically retrieves search data from the GT Explorer Tool, which is an index scaled from 0 to100, indicating the popularity of searches during the study period [15]. On a particular day, if there is insufficient RSV the value is zero, and 100 represents peak popularity [42]. The two-letter code provided by the explorer tool was used to restrict the geographical location for each country. For instance, data on Nigeria was extracted using ’NG’ as the code (see https://trends.google.com/trends/?geo=NG).

We relied on daily and weekly search data collected in June 2021 for January to August of each year (2019 and 2020), and the 2019 data acts as a counterfactual dataset. The study period coincides with the dates when schools were closed in March 2020 (Table 2 below for details). The school closure dates provided by the United Nations Educational, Scientific and Cultural Organisation (UNESCO) [20], were used instead of the lockdown dates since many SSA countries did not impose national lockdown. Furthermore, the school closure dates mostly preceded and lasted longer than the lockdown dates for countries that imposed localised or national lockdown. Using the school closure dates helps account for any psychosocial effects that may have emerged as soon as mobility restrictions were adopted. Additionally, school closures have been linked to poor mental well-being due to their effects on the daily routines of school-going individuals, their parents/caregivers, and their family members [36].

Furthermore, the study period coincided with marked concerns and changes in circumstances due to COVID-19 and changes in population circumstances [11]. To ensure comparability with the 2020 data, we applied Brodeur et al.’s, (2021) [7] scaling procedure which adjusts for seasonal fluctuations and complications due to unexplained peaks, valleys, and null searches over weeks. This entails generating weekly search interest weights, using the weights to re-scale the daily data for each by topic *i* and year *t*; *D*^¯^*i,t*, and then normalising them to a range of 0 to 100 [7].

### Measures

We selected the macro topics for the study following a review of related articles on the impact on COVID-19 on searches for mental health and subjective well-being [6–8]. The final topics were grouped into three themes: Social dysfunction (Anger, Boredom and Loneliness); Anxiety (Fear, Panic, Sleep and Worry); and Sadness (Sadness and Suicide) [6–8]. To accommodate related search terms and account for differences in upper and lower cases, as well as group words that share the same concept in any language, the study adopted topic queries instead of search terms. The advantage of using ‘topic queries’ is that it includes the semantics of the words as they cover all search terms related to the keywords [37].

In addition to the aforementioned topics, we analysed the impact of school closures on an unfiltered sample of search requests for two plausible coping mechanisms during a crisis: prayer and exercise. In the SSA region, on average, 96% of adults identified as religious: 63% identifying as Christian, 30% as Muslim, and 3% as practitioners of folk or traditional religions [38]. Research indicates that religion plays a significant role in people’s lives, particularly during times of crisis when individuals must confront unpredictable and overwhelming events like natural disasters, the death of a loved one, or life-threatening illnesses or accidents. Prayer serves as a means of finding comfort, seeking explanations, and fostering a closer relationship with God [39, 40]. For example, following the Christchurch New Zealand earthquake, individuals reported increased religious faith [41], and after the September 11, 2001 terrorist attacks, 90% of Americans reported relying on religion to cope with the aftermath [42]. Furthermore, research has shown that exercise can be a protective factor against poor mental health [43].

### Analytical strategy

***Initially***, we utilized graphical analysis to illustrate the trends in mental health and well-being-related search patterns. Following this, we employed the difference-in-difference (DiD) estimator to assess the combined impact of the COVID-19 pandemic and school closures on searches related to well-being. To account for seasonal variations within countries, we compared pre- and post-pandemic search patterns in 2020 with the same period in 2019. Finally, we conducted an event study analysis as a robustness check for the main DiD estimates and to examine the effects of population adaptation to the pandemic and the school closures. All analyses were conducted using Stata version 14 (StataCorp LLC, Texas, United States of America) and statistical significance was set at the 5% level.

#### Difference-in-differences estimator of school closures

The DiD regression model is as follows:

1 $$\eqalign{{WB}_{ i,c} & ={\alpha T}_{i,c} \times {Year}_{i}\cr & +{\beta T}_{i,c}+{\delta X}_{i-1,c}+{\mu }_{i }+{\rho }_{c }+{\epsilon}_{i,c}}$$

where *WB*_*i,c*_ denotes the RSV on day *i* for country *c*. The estimate α reflects the impact of a school closure on the RSV for topic WB on day *i* in country *c. T*_*i,c*_ is a dummy that equals *one for* days after the school closure and *zero* otherwise, while *Year*_*i*_ corresponds to 2020. Equation 1 adjusts for seasonal variations or weekly cycles using the year, week, and day fixed effects in the vector *µ*, and the country fixed effects *ρ*. The identification strategy in Eq. 1 depends on two assumptions: (1) school closures vary between the countries. and (2) the comparison of the Google search intensity (RSV) for topic *WB* before and after the school closure date in 2020 to the difference in the RSV for the same topic before and after the same date in 2019. The standard errors are robust and clustered at the day level. The variable *X*_*i−*1,*c*_ controls for COVID-19 intensity data extracted from the OxCGRT data [43], defined as the lagged number of confirmed COVID deaths per day per million in each analysed country.

The DiD approach has a significant limitation and in this study it is associated with an individual’s level of awareness of the COVID-19 pandemic. For instance, an individual may decide to distance themselves physically for reasons unrelated to NPIs, which could result in loneliness and reduced well-being. Our baseline equation’s key assumption is that, in the absence of school closures, the average Google user’s search behaviour in the sample countries would have followed the same trend observed in 2019. This means that ‘the trajectories across areas would have evolved to preserve pre-existing differences, so any subsequent deviations between areas can be attributed to the pandemic’ [44]. This assumption would be invalid if individuals residing in the countries in the sample experienced specific shocks that differ from those experienced in 2019.

### Event study estimator of school closures

The event study model is defined as follows:

2 $$\eqalign{{WB}_{ i,c} & ={\sum }_{k=-3}^{k=10}{\alpha }_{k}^{{\prime }{\prime }}{E}_{k,c}\times {Year}_{i}+{\sum }_{k=-3}^{k=10} \cr & {\beta }_{k}^{{\prime }{\prime }}{E}_{k,c}+ {\gamma }^{{\prime }{\prime }}{X}_{i-1,c}+{\mu }_{i}^{{\prime }{\prime }}+{\rho }_{c}^{{\prime }{\prime }}+{\epsilon}_{i}^{{\prime }{\prime }}}$$

where ${E}_{k,c}$ is a set of dummy variables that takes the value of one for the three weeks before the schools were closed in each country and the ten weeks after (which is interacted with the dummy variable ${Year}_{i}$ for the year 2020). The reference period is the fourth week before the school closures and takes the value of zero. The ${E}_{k,c}$ dummies are the estimated coefficients and indicate the effect of being in a particular week after school were closed (for example, the sixth week, (${}_{6,c}$) compared to four weeks before. Equation 2 also adjusts for the year, week, and day fixed effects in vector ${\mu }_{i}^{{\prime }{\prime }}$ to account for seasonal effects, and the country fixed effects ${\rho }_{c}^{{\prime }{\prime }}$ that consider for time-invariant differences across the countries.

## Results

### Graphical analysis

To illustrate the pre- and post-school closure search patterns, figuresS1-S4 (see supplementary material) show the raw daily searches for the topics 56 days before and 28 days after schools closed due to the pandemic. The red and black lines represent the average searches for 2020 and 2019, respectively. Overall, we observed considerable variation in daily searches over time. The RSVs for anger, boredom and prayer increased sharply around the time of school closures in 2020. The figures also display noticeable increases in anxiety-related topics, and fear peaked in the second week of school closures. However, the uptick in panic began two weeks before the school closures. Regarding sleep and worry, the results suggest that the RSV began to rise around the school closure dates but peaked at different times. Specifically, the RSV for sleep peaked in the second week after school closures, whereas searches for worry reached their highest point at the end of the first week.

The search for sadness peaked in the second week of school closures, while no clear patterns were observed for the raw searches related to suicide. Finally, searches for exercise saw an uptick in the second week of school closures and peaked at the end of the third week following the school closures.

### Difference-in-differences estimation results

Figure S5 (See supplementary material) depicts the estimated effects, while Table 3 presents coefficients. The findings indicate a significant increase in the RSV of anger and boredom at the 1% level. The model coefficients in Table 3 suggest that the RSV for ‘Boredom’ was approximately 10 times higher (*α* = 9.64, p < 0.001) post-school closure, compared to the pre-school closure period. School closures were also significantly and positively associated with the relative search volume for ‘Anger’. The RSV for anger was, on average, 6.7 times higher (*α* = 6.65, p < 0.001) post-school closure, relative to the pre-school closure period. There was no evidence of an association between school closures and the relative search volume of loneliness.

The results for anxiety-related topics (fear, panic, sleep and worry)are mixed. The coefficients for fear (*α* = 5.18, p < 0.001) and sleep (*α* = 1.93, p < 0.001) are positive and statistically different from zero, while the coefficient for panic (*α* = -2.87, p < 0.001) is negative and statistically significant. There was no evidence of an association between school closures and the relative search volume for ‘Worry’. Additionally, the coefficients for sadness and suicide are positive but statistically insignificant. Finally, the results show a positive and significant association between school closures and the search intensity for the coping-related topics: prayer and exercise. The RSV for prayer was approximately 4 times higher (p < 0.001) post-school closure, relative to the pre-school closure period. For exercise, the RSV was on average 2.2 times higher (p < 0.001) post-school closure, relative to the pre-school closure period.

**Table 3:** The Effects of School Closures - DiD Estimates (Figs. S1-S4).

Panel A: Social Dysfunction				
	Anger	Boredom	Loneliness	
Tic*Yeari	6.65***	9.64***	0.90	
	(1.28)	(1.43)	(1.51)	
Country FE	Yes	Yes	Yes	
Year, Week and Day FE	Yes	Yes	Yes	
Deaths	Yes	Yes	Yes	
Observations	2230	756	583	
Panel B: Anxiety				
	Fear	Panic	Sleep	Worry
Tic*Yeari	5.18***	-2.87***	1.93***	-1.10
	(1.18)	(0.87)	(0.35)	(0.73)
Country FE	Yes	Yes	Yes	
Year, Week and Day FE	Yes	Yes	Yes	Yes
Deaths	Yes	Yes	Yes	Yes
Observations	3183	465	7897	1099
Panel C: Sadness and Coping				
	Sadness	Suicide	Exercise	Prayer
Tic*Yeari	0.24	0.05	2.22***	4.32***
	(0.60)	(0.22)	(0.27)	(0.53)
Country FE	Yes	Yes	Yes	
Year, Week and Day FE	Yes	Yes	Yes	Yes
Deaths	Yes	Yes	Yes	Yes
Observations	3019	1519	6088	9213

Notes: This table presents the results of the differences-in-differences (DiD) estimates. All models include control for the number of days elapsed after the closure of schools, as well as the time (year, week, day of the week) fixed effects and the one- day lagged number of deaths from COVID-19 per million. Standard errors are clustered at the day level and are in parentheses. Statistical significance is denoted by: *** p < 0.01, ** p < 0.05, * p < 0.1

As previously stated, to test the sensitivity of the estimates, we restrict the sample to 13 countries (Angola, Eswatini, Ethiopia, Guinea, Kenya, Liberia, Nigeria, Republic of Congo, Sudan, The Gambia, Uganda, Zimbabwe) with high death rates per million population and high average stringency index. We find that the results are quantitatively similar (Fig.S4 and Table 4 below). It is not surprising that the the estimates for anger, boredom, fear, panic, exercise, and prayer are larger, and similar conclusions can be drawn regarding the effects of school closures on well-being-related Google searches.

**Table 4:** The Effects of School Closure Order - DiD Estimates (Robustness Checks)

Panel A: Social Dysfunction				
Variable	Anger	Boredom	Loneliness	
Tic*Yeari	8.22***	10.09***	1.12	
	(1.52)	(1.66)	(1.63)	
Country FE	Yes	Yes	Yes	
Year, Week and Day FE	Yes	Yes	Yes	
Deaths	Yes	Yes	Yes	
Observations	1310	510	427	
Panel B: Anxiety				
Variable	Fear	Panic	Sleep	Worry
Tic*Yeari	6.51***	-2.54***	1.77***	-1.09
	(1.47)	(0.93)	(0.40)	(0.78)
Time FE	Yes	Yes	Yes	Yes
Deaths	Yes	Yes	Yes	Yes
Observations	1667	337	3567	789
Panel C: Sadness and Coping				
Variable	Sadness	Suicide	Exercise	Prayer
Tic*Yeari	-0.49	0.00	2.58***	5.02***
	(0.67)	(0.27)	(0.36)	(0.72)
Time FE	Yes	Yes	Yes	Yes
Deaths	Yes	Yes	Yes	Yes
Observations	1585	1005	3091	3785

Notes: This table presents the results of the differences-in-differences (DiD) estimates for countries that had high average stringency index during the study period (0.50). The 13 countries are: Angola, Nigeria, Kenya, Zimbabwe, Republic of Congo, Eswatini (Swaziland), Uganda, Guinea, Ethiopia, Liberia, Sudan, and The Gambia. All models include control for the number of days elapsed after the closure of schools, as well as the time (year, week, day of the week) fixed effects and the one- day lagged number of deaths from COVID-19 per million. Standard errors are clustered at the day level and are in parentheses. Statistical significance is denoted by: *** p < 0.01, ** p < 0.05, * p < 0.1

### Event study results

As explained above, we conducted an event study analysis to examine adaptation to the pandemic and the associated school closures. The results are presented in Figures S6, S7 and S8 (See supplementary material) for social dysfunction, anxiety, sadness, and coping, respectively. The findings suggest that the effects of school closures on well-being related topics are mixed. For instance, searches for boredom began to rise before the school closures and lasted for about five weeks. Additionally, the RSV for fear increased weeks before the school closures but began to decline at the school closure and dropped to zero by the fifth week (see the first graph in figure S7). The searches for exercise began to rise in the week before the school closures, and remained above the baseline level for 10 weeks post-school closure. Finally, the effects on prayer began to rise before the school closures and remained higher than the baseline level for 7 weeks following the school closures.

### Discussion of findings

In response to the threat of the COVID-19 pandemic, governments worldwide implemented school closures as part of mobility restrictions. This study utilised multiple approaches to estimate the impact of the pandemic-induced school closures on mental health and well-beings, as well as coping strategies in selected Sub-Saharan Africa (SSA) countries. We quantified this impact by examining changes in the search volume for topics related to three themes on Google Trends (GT): Social dysfunction (Anger, Boredom and Loneliness), Anxiety (Fear, Panic, Sleep and Worry), and Sadness (Sadness and Suicide). Searches for two coping mechanisms, namely exercise and prayer, were also included in the analysis. We leveraged the availability of GT data, and the temporal and cross-sectional variation in school closures to provide a unique opportunity to estimate the population’s responses to school closures.

The study findings produced mixed results. On one hand, pandemic-induced school closures had a positive and statistically significant effect on the search volume for anger, boredom, fear, sleep, prayer and exercise. The event-study results indicated that the higher search volume lasted for several weeks following the school closures. For example, increased searches for boredom and fear persisted for approximately five weeks, while those for prayer lasted for seven weeks, and exercise lasted for about 10 weeks. On the other hand, we observed significantly lower searches for panic. Finally, we find no discernible evidence of an effect of school closures on topics related to loneliness, sadness, suicide, worry.

Previous studies that tracked temporal changes in health and well-being-related topics before and during the pandemic’s mobility restrictions [6–8, 42, 45] also noted higher search volumes for topics related to boredom and anger. If we assume that Google users search topics based on their mood, the findings suggest that the population was more bored during the school closures. However, over time, these searches decreased which could be due to adaptation to the “new normal” and the introduction of online learning and working strategies adopted in different countries [7, 20]. Anger is also a basic emotion experienced during the pandemic. It has been noted in the literature that the fear state (fear of the disease and fear of change due to the disease) which was evident during the pandemic, can trigger anger [46]. For instance, the lack of proper communication and education about NPIs (e.g., wearing of face masks while outdoors) may have caused arguments and confrontation regarding political control of people’s liberties [47].

The lack of discernible effects of school closures on loneliness could be attributed to the availability of social media platforms (such as Facebook, Instagram, TikTok, and YouTube) and online messaging platforms (such as WhatsApp), which may have allowed for frequent virtual contact with family and friends, as well as entertainment. These virtual means of staying connected and receiving emotional support may have mitigated the negative effects of the pandemic, reducing the impact on loneliness [48]. The presence of social media may have presented the opportunity for individuals to find creative ways to fulfil their need for social connection.

It is not surprising that school closures had no significant impact on the relative search volume for sadness and suicide. Like loneliness, the presence of social media and online messaging platforms may have reduced the impact of the physical restrictions on sadness, thus decreasing the likelihood of suicidal ideation or thoughts. In addition to virtual social contact, the sense of a “coming together” phenomenon during the crisis, the availability of financial safety nets, and the decline in the monetary and psychosocial costs of commuting, especially for poorer individuals who travel long distances to school and work, may have moderated the negative effects of COVID-19 [49, 50].

The finding of a positive effect on prayer is consistent with other studies conducted in countries with a higher prevalence of religious populations, including those with large populations of Christians and Muslims, as well as countries where religion is practised sporadically or not at all [51, 52]. Prayer can have a positive effect on mental health and improve well-being by helping people cope with stressful situations, thereby reducing anxiety and tension [53]. This effect is particularly pronounced for individuals who regularly confront adversity [51]. Bentzen [51] noted that during times of adversity or uncertainty, when people need to cope, their use of religion is mainly intrinsic (such as private prayers for relief, understanding, and comfort) rather than extrinsic, such as attendance at religious institutions like churches. However, comparing these motivations and determining whether the rise in searches for prayer is permanent is beyond the scope of this study.

The results for the higher search volume for fear confirm the finding of other studies that the pandemic and the NPIs introduced to curb the transmission of the coronavirus introduced anxiety, especially at the early stages of the pandemic [7, 11]. The increased searches for fear could be attributed to several factors including the fear of contracting or having a loved one infected with COVID-19, as well as concerns about economic insecurity and future prospects [6, 54]. Fear-related searches by the population began before the school closures were implemented, which is consistent with the findings of other studies [45, 55]. The literature suggests that fear surrounding infection began to increase shortly after COVID-19 was declared a pandemic in the absence of an effective treatment, and began to decrease as mobility restrictions were imposed and people adapted psychologically to the presence of COViD-19 [45, 55].

Similarly, the increase in panic-related searches began before the implementation of school closures, which can be attributed to the observation of the pandemic’s development in other countries and the panic-buying that arose as people planned for possible mobility restrictions [56]. The heightened searches for sleep and exercise may be linked to the increased boredom and fear among the population. Given the sudden changes in daily life and more time on their hands, individuals may increase their online searches, looking for guidance on improving sleep quality or including forms of exercise to include into their daily routine [57], or have become more aware of the benefits of sleep and exercise for physical and mental health [58].

### Strengths and limitations

This study utilised data from GT® to investigate the association between COVID-19 pandemic-induced school closures in SSA countries and searches for key mental health and subjective well-being topics. GT® provides an alternative to traditional data sources as it provides another source of data. The data can be seamlessly exported into a comma separated value (.csv) file and imported into analytical software such as Stata for analysis. Although imperfect, the GT® data allows researchers to use big data and circumvent some of the biases that are common in survey data, such as self-reporting bias (where survey respondents may not disclose the truth), recall bias (due to the time lag between the event and the survey), and social desirability bias (due to perceived interviewer-expectations). Another key advantage of the GT® data is that it allows researchers to document public responses and examine the effects of the COVID-19 measures on well-being at a time when data collection was expensive, and participants were harder to reach. Moreover, it allows for the exploration of associations between mobility restrictions and mental health and well-being for a large group of countries.

Despite the benefits, there are limitations that warrant discussion. First, the nature of the data does not allow heterogeneity based on the searchers’ sociodemographic characteristics, such as age, sex, housing conditions, educational level, occupation, perceived risk of infection, and access to mental healthcare services. Second, the sample is not representative of the population, as most of the internet users in the region are young and relatively well-off, those who can afford smart devices and internet data costs. Thus, the results may be skewed towards predicting the searches of this group and ignore vulnerable groups, such as the poor, elderly, and those living with pre-existing mental health issues or disabilities who may not be active searchers. Third, the data does not efficiently account for unique social, political, and economic realities that present diverse experiences within and across the countries under investigation.

## Conclusion

The COVID-19 outbreak posed a significant threat to population health and well-being. This paper assessed the association between COVID-19 school closures and searches for anger, boredom, loneliness, fear, panic, sleep, worry, sadness, suicide, exercise, and prayer in selected countries of Sub-Saharan Africa using GT® data. The study provides evidence demonstrating the potential for GT® data to be leveraged as a data source for understanding how populations across countries reacted to the COVID-19 pandemic and the resultant social policies. Although the increases in the relative search volumes of several well-being related and coping topics (anger, boredom, fear, sleep, exercise, and prayer) may not necessarily indicate individuals developing long-term mental distress and poor subjective well-being, they highlight the importance of increased vigilance and proactive communication from the government and policy makers to the general population. These are necessary in emergencies when social policies that restrict lives, livelihoods and liberties must be adopted. The study’s findings should be interpreted as the average impact of the mobility restrictions on the well-being interests of Google Search users pre- and post–school closures in 2020 to the same period in 2019, and not taken as a ‘window into the soul’ of the population. It is important that SSA countries collect detailed surveys on mental health and well-being, which can be triangulated with GT® data to provide reliable evidence for policies on population health and well-being.

## Electronic supplementary material

Below is the link to the electronic supplementary material.

Supplementary Material 1

## Data Availability

The datasets generated and/or analysed during the current study are publicly available in https://trends.google.com/trends/?geo=NG In addition, human subjects were not directly involved in this study.

## List of Abbreviations

CSV: CommaSeparated Value
DiD: Difference-in-Difference
GT: Google Trends
NPI: Non-pharmaceutical Interventions
OxCGRT: Oxford COVID-19 Government Response Tracker
SSA: Sub-Saharan Africa
UNESCO: United Nations Educational, Scientific and Cultural Organisation

## References

[1] Mangono T, Smittenaar P, Caplan Y, Huang VS, Sutermaster S, Kemp H et al. Information-seeking patterns during the COVID-19 pandemic across the United States: Longitudinal analysis of Google trends data. J Med Internet Res [Internet]. 2021;23(5):e22933. Available from: 10.2196/22933. 10.2196/22933 PMC8095345 33878015

[2] Amare M, Abay KA, Tiberti L, Chamberlain J, Impact. of COVID-19 on food security: Panel data evidence from Nigeria. IFRPI Discussion Paper. [Internet]. Washington DC; 2020. Report No.: 1956. Available from: 10.2499/p15738coll2.133866.

[3] Mohammed O, Miriri D. African nations close borders, cancel flights to contain coronavirus spread. [Internet]. London; 2020. Available from: https://www.reuters.com/article/us-health-coronavirus-africa-idUSKBN2120YR.

[4] Kallander SW, Gordon R, Borzekowski DLG. “People will continue to suffer if the virus is around”: A qualitative analysis of Sub-Saharan African children’s experiences during the COVID-19 pandemic. Int J Environ Res Public Health [Internet]. 2021;18(11):5618. Available from: 10.3390/ijerph18115618. 10.3390/ijerph18115618 PMC8197409 34070258

[5] Alfano V, Ercolano S, Vecchione G, Religious attendance. and COVID-19. evidence from Italian religions. [Internet]. Munich; 2020. Report No.: 8596. Available from: https://ideas.repec.org/p/ces/ceswps/8596.html.

[6] Boateng GO, Doku DT, Enyan NIE, Owusu SA, Aboh IK, Kodom RV et al. Prevalence and changes in boredom, anxiety and well-being among Ghanaians during the COVID-19 pandemic: A population-based study. BMC Public Health [Internet]. 2021;21(985). Available from: 10.1186/s12889-021-10998-0. 10.1186/s12889-021-10998-0 PMC8149916 34039313

[7] Brodeur A, Clark AE, Fleche S, Powdthavee N. COVID-19, lockdowns and well-being: Evidence from Google trends. J Public Econ [Internet]. 2021;193(3):104346. Available from: 10.1016/j.jpubeco.2020.104346. 10.1016/j.jpubeco.2020.104346 PMC7703221 33281237

[8] Foa RS, Gilbert S, Fabian MO. COVID-19 and subjective well-being: Separating the effects of lockdowns from the pandemic. [Internet]. Cambridge; 2020. Available from: https://www.bennettinstitute.cam.ac.uk/wp-content/uploads/2020/12/Happiness under Lockdown.pdf.

[9] Halford EA, Lake AM, Gould MS. Google searches for suicide and suicide risk factors in the early stages of the COVID-19 pandemic. PLoS One [Internet]. 2020;15(7):e236777. Available from: 10.1371/journal.pone.0236777. 10.1371/journal.pone.0236777 PMC7380602 32706835

[10] International Organisation for Migration. Mental health and psychological support (MHPSS) in the COVID-19 response: Guidance and toolkit for the use of IOM MHPSS teams - Version III-final. [Internet]. Geneva. ; 2020. Available from: https://www.iom.int/sites/g/files/tmzbdl486/files/documents/mhpss-covid-19-guidance-toolkit-v3-en.pdf.

[11] Knipe D, Evans H, Marchant A, Gunnell D, John A. Mapping population mental health concerns related to COVID-19 and the consequences of physical distancing: A Google trends analysis. Wellcome Open Res [Internet]. 2020;5(82):1–17. Available from: 10.12688/wellcomeopenres.15870.2. 10.12688/wellcomeopenres.15870.1 PMC7331103 32671230

[12] Pierce M, Hope H, Ford T, Hatch S, Hotopf M, John A et al. Mental health before and during the COVID-19 pandemic: A longitudinal probability sample survey of the UK population. Lancet Psychiatry [Internet]. 2020;7(10):883–92. Available from: 10.1016/S2215-0366(20)30308-4. 10.1016/S2215-0366(20)30308-4 PMC7373389 32707037

[13] Banks J, Fancourt D, Xiaowei X. Mental health and the COVID-19 pandemic. In: Helliwell JF, Layard R, Sachs JD, De Neve J-E, Aknin LB, Wang S, editors. World Happiness Report [Internet]. 2021st ed. New York: Sustainable Development Solutions Network; 2021. Available from: https://happiness-report.s3.amazonaws.com/2021/WHR+21.pdf.

[14] Timoneda JC, Vera SV. Will I die of coronavirus? Google trends reveal that politics determine virus fears. PLoS One [Internet]. 2020;16(10):e0258189. Available from: 10.1371/journal.pone.0258189. 10.1371/journal.pone.0258189 PMC8494313 34614032

[15] Berger LM, Ferrari G, Letrurcq M, Panico L, Solaz A. COVID-19 lockdowns and demographically-relevant Google trends: A cross-national analysis. PLoS One [Internet]. 2021;16(3):e0248072. Available from: 10.1371/journal.pone.0248072. 10.1371/journal.pone.0248072 PMC7968661 33730055

[16] Cucinotta D, Vanelli M, WHO Declares COVID-19 a Pandemic. Acta Biomed [Internet]. 2020;91(1):157–60. Available from: 10.23750/abm.v91i1.9397. 10.23750/abm.v91i1.9397 PMC7569573 32191675

[17] Worldometers COVID. Live - Coronoavirus Statistics [Internet]. Shanghai; 2021. Available from: http://www.worldometers.info/coronavirus/.

[18] Letzing J, What. does COVID-19 still have in store for Africa? [Internet]. Geneva; 2020. Available from: https://www.weforum.org/agenda/2020/04/coronavirus-what-does-covid-19-still-have-in-store-for-africa/.

[19] Ritchie H, Mathieu ER-G, Appel C, Giattino C, Ortiz-Ospina E, Hasell J et al. Coronavirus Pandemic (COVID-19). Our World in Data. [Internet]. 2020. Available from: https://ourworldindata.org/coronavirus.

[20] UNESCO Institute for Statistics. COVID-19 education response [Internet]. Quebec. ; 2020. Available from: http://data.uis.unesco.org.

[21] Dunford D, Dale B, Stylianou N, Lowther E, Ahmed M, Arenas I, Coronavirus. The world in lockdown in maps and charts. [Internet]. London; 2020. Available from: https://www.bbc.co.uk/news/world-52103747.

[22] The Guardian. First death from China mystery illness outbreak. 2020; Available from: https://www.theguardian.com/world/2020/jan/11/china-mystery-illness-outbreak-causes-first-death.

[23] World Health Organisation. Novel Coronavirus (2019-nCoV): situation report, 3 [Internet]. Geneva; 2020. Available from: https://apps.who.int/iris/handle/10665/330762.

[24] Tian H, Liu Y, Li Y, Wu CH, Chen B, Kraemer M et al. An investigation of transmission control measures during the first 50 days of the COVID-19 epidemic in China. Science (80-) [Internet]. 2020;368(6491):638–42. Available from: 10.1126/science.abb6105. 10.1126/science.abb6105 PMC7164389 32234804

[25] World Health Organisation. Mental health: Strengthening our response. [Internet]. Geneva. ; 2018. Available from: https://www.who.int/news-room/fact-sheets/detail/mental-health-strengthening-our-response.

[26] Nyundo A, Manu A, Regan M, Ismail A, Chukwu A, Dessie Y et al. Factors associated with depressive symptoms and suicidal ideation and behaviours amongst sub-Saharan African adolescents aged 10–19 years: cross-sectional study. Trop Med Int Health [Internet]. 2020;25(1):54–69. Available from: 10.1111/tmi.13336. 10.1111/tmi.13336 31698526

[27] Mazwi N, Seremani B, Kaseke T, Lungu C. Psycho-Social experiences of youths during the COVID-19 lockdown: Insights from Harare, Zimbabwe. Bus Excell Manag [Internet]. 2020;10(5):46–59. Available from: https://econpapers.repec.org/RePEc:rom:bemann:v:10:y:2020:i:5:p:46-59.

[28] Rother H-A, Etzel RA, Shelton M, Paulson JA, Hayward RA, Theron LC. Impact of extreme weather events on Sub-Saharan African child and adolescent mental health: A protocol for a systematic review. Atmosphere (Basel) [Internet]. 2020;11(5):493. Available from: 10.3390/atmos11050493.

[29] Ahorsu DK, Lin C-Y, Imani V, Saffari M, Griffiths MD, Pakpour AH. The fear of COVID-19 Scale: Development and initial validation. Int J Ment Health Addict [Internet]. 2020;20(3):1537–45. Available from: 10.1007/s11469-020-00270-8. 10.1007/s11469-020-00270-8 PMC7100496 32226353

[30] Honermann BJ. An “Epidemic within an outbreak”: The mental health consequences of infectious disease epidemics. [Internet]., Washington DC. ; 2015. Available from: https://oneill.law.georgetown.edu/epidemic-within-outbreak-mental-health-consequences-infectious-disease-epidemics/.

[31] International Labour Organisation, Youth. & COVID-19: Impacts on Jpbs, education, rights and mental well-being [Internet]. 2020 [cited 2020 Sep 12]. p. 1–48. Available from: https://www.ilo.org/wcmsp5/groups/public/---ed_emp/documents/publication/wcms_753026.pdf.

[32] Mahmud M, Riley E. Household response to an extreme shock: Evidence on the immediate impact of the Covid-19 lockdown on economic outcomes and well-being in rural Uganda. World Dev [Internet]. 2021;140(105318). Available from: 10.1016/j.worlddev.2020.105318. 10.1016/j.worlddev.2020.105318 PMC8446716 34548741

[33] Stuijfzand S, Deforges C, Sandoz V, Sajin CT, Jaques C, Elmers J et al. Psychological impact of an epidemic/pandemic on the mental health of healthcare professionals: A rapid review. BMC Public Health [Internet]. 2020;20(1):1230. Available from: 10.1186/s12889-020-09322-z. 10.1186/s12889-020-09322-z PMC7422454 32787815

[34] Aya C. COVID-19 in Africa: Youth perspectives. [Internet]. London; 2020. Available from: https://mo.ibrahim.foundation/news/2020/covid-19.

[35] Office for National Statistics UK. Coronavirus and anxiety, Great Britain. [Internet]. Newport. ; 2020. Available from: https://www.ons.gov.uk/peoplepopulationandcommunity/wellbeing/articles/coronavirusandanxietygreatbritain.

[36] UNFPA & IFRC. COVID-19: Working with and for young people [Internet]. New York. ; 2020. Available from: https://www.unfpa.org/resources/covid-19-working-and-young-people.

[37] Javed B, Sarwer A, Soto EB, Mashwani ZU. The coronavirus (COVID-19) pandemic’s impact on mental health. Int J Heal Plan Manag [Internet]. 2020;35(5):993–6. Available from: 10.1002/hpm.3008. 10.1002/hpm.3008 PMC7361582 32567725

[38] Johal SS. Psychosocial impacts of quarantine during disease outbreaks and interventions that may help to relieve strain - PubMed. N Z Med J [Internet]. 2009 [cited 2020 Sep 12];122(1296):47–52. Available from: https://pubmed.ncbi.nlm.nih.gov/19652680/. 19652680

[39] African Union. Member States. [Internet]. 2022. Available from: https://au.int/en/member_states/countryprofiles2.

[40] Massicotte P, Eddelbuettel D. GtrendsR: R functions to perform and display Google trends queries. [Internet]. 2016. Available from: https://cran.r-project.org/package=gtrendsR.

[41] R Core Team. R: A language and environment for statistical computing [Computer Software]. [Internet]. 2019. Available from: https://www.r-project.org/.

[42] Silverio SA, De Backer K, Easter A, von Dadelszen P, Magee LA, Sandall J. Women’s experiences of maternity service reconfiguration during the COVID-19 pandemic: A qualitative investigation. Midwifery [Internet]. 2021;102:103116. Available from: 10.1016/j.midw.2021.103116. 10.1016/j.midw.2021.103116 PMC9756856 34399382

[43] Hale T, Angrist N, Goldszmidt R, Kira B, Petherick A, Phillips T et al. A global panel database of pandemic policies (Oxford COVID-19 Government Response Tracker). Nat Hum Behav [Internet]. 2021;5:529–38. Available from: 10.1038/s41562-021-01079-8. 10.1038/s41562-021-01079-8 33686204

[44] Banks J, Xiaowei X. The mental effects of the first two months of lockdown and social distancing during the COVID-19 pandemic in the UK. Fisc Stud [Internet]. 2020;41(3):685–708. Available from: 10.1111/1475-5890.12239.

[45] de la Rosa PA, Cowden RG, de Filippis R, Jerotic S, Nahidi M, Ori D et al. Associations of lockdown stringency and duration with Google searches for mental health terms during the COVID-19 pandemic: A nine-country study. J Psychiatry Res [Internet]. 2022;150:237–45. Available from: 10.1016/j.jpsychires.2022.03.026. 10.1016/j.jpsychires.2022.03.026 PMC8971703 35398667

[46] Zhan J, Ren J, Sun P, Fan J, Liu C, Luo J. The neural basis of fear promotes anger and sadness counteracts anger. Neural Plast [Internet]. 2018;3479059. Available from: 10.1155/2018/3479059. 10.1155/2018/3479059 PMC6022272 30013595

[47] Rahman M, Ahmed R, Moitra M, Damschroder L, Brownson R, Chorpita B et al. Mental Distress and Human Rights Violations during COVID-19: a Rapid Review of the evidence informing rights, Mental Health needs, and Public Policy around vulnerable populations. Front Psychiatry. 2021;11(January). 10.3389/fpsyt.2020.603875 PMC7820171 33488426

[48] Luchetti M, Lee JH, Aschwanden D, Sesker A, Strickhouser JE, Terracciano A et al. The trajectory of loneliness in response to COVID-19. Am Psychol [Internet]. 2020;75(7):897–908. Available from: 10.1037/amp0000690. 10.1037/amp0000690 PMC7890217 32567879

[49] Sinyor M, Spittal MJ, Niederkrotenthaler T. Changes in suicide and resilience-related Google searches during the early stages of the COVID-19 pandemic. Can J Psychiatry [Internet]. 2020;65(10):741–3. Available from: 10.1177/0706743720933426. 10.1177/0706743720933426 PMC7502879 32524848

[50] University of Cambridge. Lockdown led to happiness rebound, after wellbeing plunged with onset of pandemic. [Internet]. Cambridge. ; 2020. Available from: https://www.cam.ac.uk/research/news/lockdown-led-to-happiness-rebound-after-wellbeing-plunged-with-onset-of-pandemic.

[51] Bentzen JS. In crisis, we pray: Religiosity and the COVID-19 pandemic. J Econ Behav Organ [Internet]. 2021;192:541–83. Available from: 10.1016/j.jebo.2021.10.014. 10.1016/j.jebo.2021.10.014 PMC8557987 34744223

[52] Boguszewski R, Makowska M, Bozewicz M, Podkowinska M. The COVID-19 Pandemic’s Impact on Religiosity in Poland. Religious [Internet]. 2020;11(12):646. Available from: 10.3390/rel11120646.

[53] Wachholtz AB, Sambamthoori U. National trends in prayer use as a coping mechanism for depression: Changes from 2002 to 2007. J Relig Health [Internet]. 2013;52(4):1356–68. Available from: 10.1007/s10943-012-9649-y. 10.1007/s10943-012-9649-y PMC4123323 23054479

[54] Duby Z, Bunce B, Fowler C, Bergh K, Jonas K, Dietrich JJ et al. Intersections between COVID-19 and socio-economic mental health stressors in the lives of South African adolescent girls and young women. Child Adolesc Psychiatry Ment Health [Internet]. 2022;16(1):23. Available from: 10.1186/s13034-022-00457-y. 10.1186/s13034-022-00457-y PMC8959551 35346316

[55] Lin YH, Chiang TW, Lin YL. Increased internet searches for insomnia as an indicator of global mental health during the COVID-19 pandemic: Multinational longtitudinal study. PLoS One [Internet]. 2020;8(12):e81422. Available from: 10.2196/22181. 10.2196/22181 PMC7508633 32924951

[56] Du H, Yang J, King RB, Chi P, Yang L. COVID-19 increases online searches for emotional and health-related terms. Appl Psychol Heal wellbeing [Internet]. 2020;12(3):1039–53. Available from: 10.1111/aphw.12237. 10.1111/aphw.12237 PMC7675240 33052612

[57] Ding D, Cruz BD, Green MA, Bauman AE. Is the COVID-19 lockdown nudging people to be more active: a big data analysis. Br J Sports Med [Internet]. 2020;54(20):1–6. Available from: 10.1136/bjsports-2020-102575. 10.1136/bjsports-2020-102575 32605932

[58] Vanguard. COVID-19 increased Nigerians’ health awareness. 2020; Available from: https://www.vanguardngr.com/2020/10/covid-19-increased-nigerians-health-awareness/.

