# Supplementary material for: School closures and well-being-related topic searches on Google during the COVID-19 pandemic in Sub-Saharan Africa
Source: BMC Public Health. 2023 Jun 27;23:1248. doi: 10.1186/s12889-023-16186-6 (PMC10294314; doi:10.1186/s12889-023-16186-6)
Supplement: Supplementary file 1 — Supplementary Material 1 [file 12889_2023_16186_MOESM1_ESM.docx]

**School closures and well-being-related topic searches on Google during the COVID-19 pandemic in Sub-Saharan Africa.**

**Supplementary Materials**

**Figure S1.** Google Search Trends in anger, boredom, and loneliness before and after the school closure.


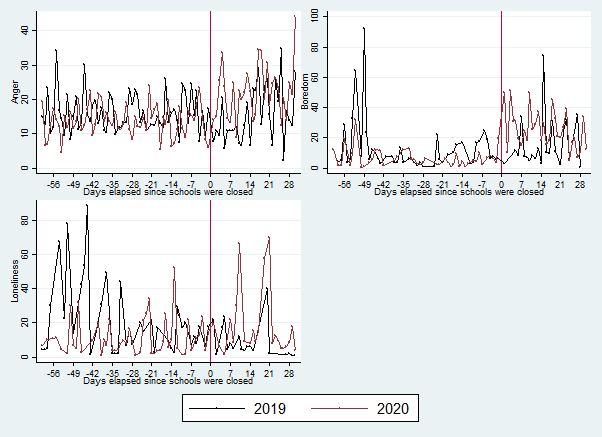


**Note:** The vertical axis shows the raw daily average scaled searches in the days before (negative values) and after (positive values) the school closure orders were initiated (set equal to day zero) in 2020 (red dots) and the same date in 2019 (black dots). The dots correspond to the raw averages by one day bins and are weighted by the population per country.

**Figure S2.** Google Search Trends in fear, panic, sleep and worry before and after the school closure.


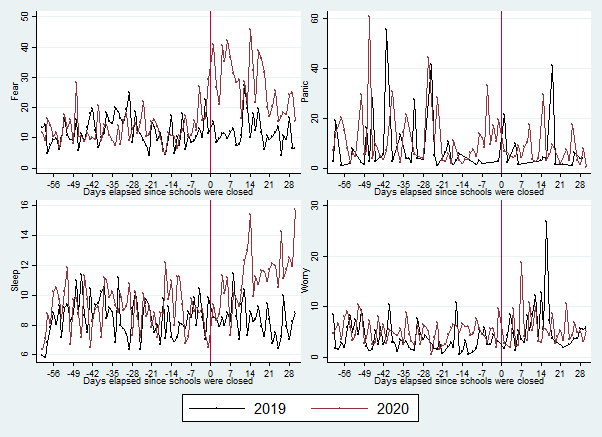


**Note:** The vertical axis shows the raw daily average scaled searches in the days before (negative values) and after (positive values) the school closure orders were initiated (set equal to day zero) in 2020 (red dots) and the same date in 2019 (black dots). The dots correspond to the raw averages by one day bins and are weighted by the population per country.

**Figure S3.** Google Search Trends in sadness and suicide before and after the school closure.


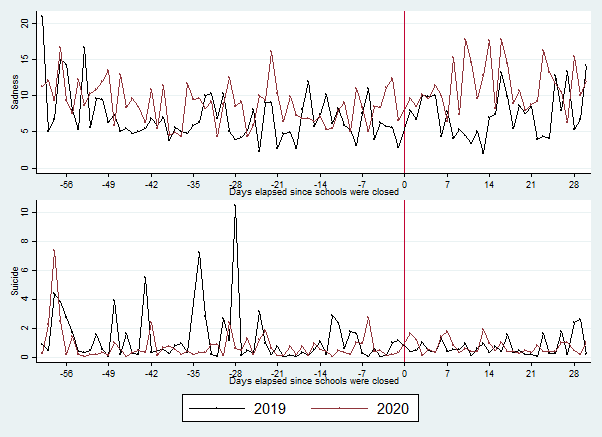


**Note:** The vertical axis shows the raw daily average scaled searches in the days before (negative values) and after (positive values) the school closure orders were initiated (set equal to day zero) in 2020 (red dots) and the same date in 2019 (black dots). The dots correspond to the raw averages by one day bins and are weighted by the population per country.

**Figure S4.** Google Search Trends in exercise and prayer before and after the school closure orders.


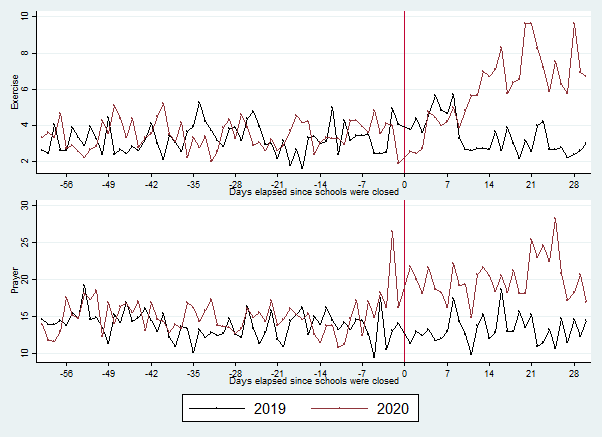


**Note:** The vertical axis shows the raw daily average scaled searches in the days before (negative values) and after (positive values) the school closure orders were initiated (set equal to day zero) in 2020 (red dots) and the same date in 2019 (black dots). The dots correspond to the raw averages by one day bins and are weighted by the population per country.

**Figure S5.** The effects of school closures on well-being and coping strategies in sub-Saharan Africa.


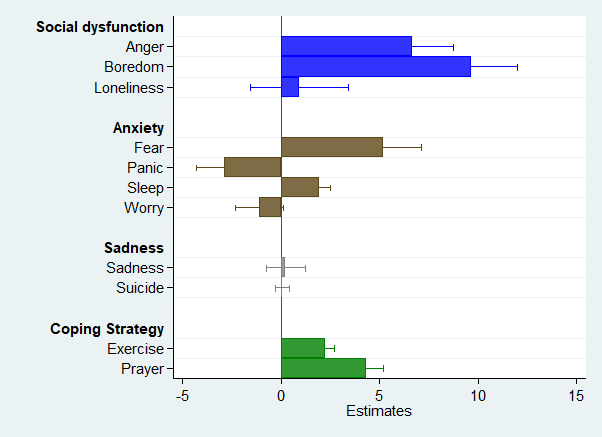


**Note:** Each bar represents regression estimates using 2019 as the counterfactual. All models include controls for the days after the announcement was made, as well as the year, week, day of the week fixed effects and the one-day lagged number of new COVID-19 cases per million. Robust standard errors and weights are included. Standard errors clustered are the day level.

**Figure S6.** Duration of the effects of school closure orders on anger, boredom, and loneliness.


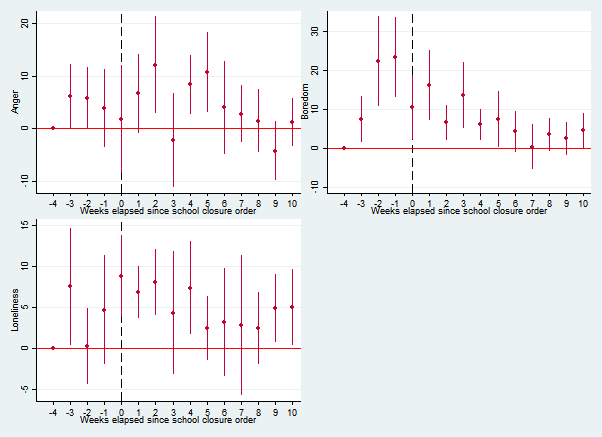


**Note:** The vertical axis shows the event-study estimates using the 2019 period as the counterfactual. The 4th week before the school closure (in 2019 or 2020) is the reference period. The models include dummies for each week from three weeks before to 10 weeks after the school closure order. In addition, controls were added for the country, year, week, and day of the week fixed effects as well as the one-day lagged number of new COVID-19 related deaths per million. Weights are applied and the standard errors which are clustered at the day level are plotted.

**Figure S7.** Duration of the effects of school closure orders on fear, panic, sleep, and worry.


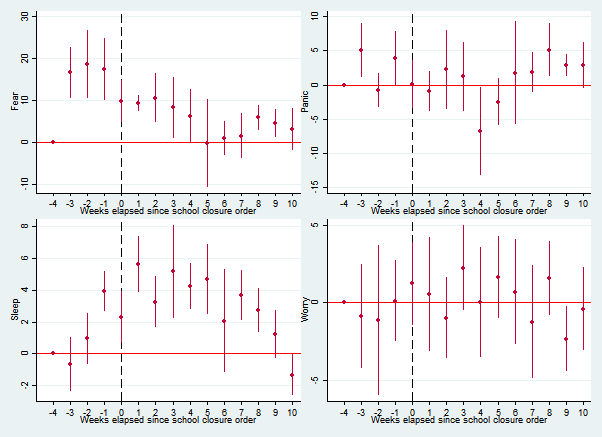


**Note:** The vertical axis shows the event-study estimates using the 2019 period as the counterfactual. The 4th week before the school closure (in 2019 or 2020) is the reference period. The models include dummies for each week from three weeks before to 10 weeks after the school closure order. In addition, controls were added for the country, year, week, and day of the week fixed effects as well as the one-day lagged number of new COVID-19 related deaths per million. Weights are applied and the standard errors which are clustered at the day level are plotted.

**Figure S8.** Duration of the effects of school closure orders on sadness, suicide, exercise, and prayer.


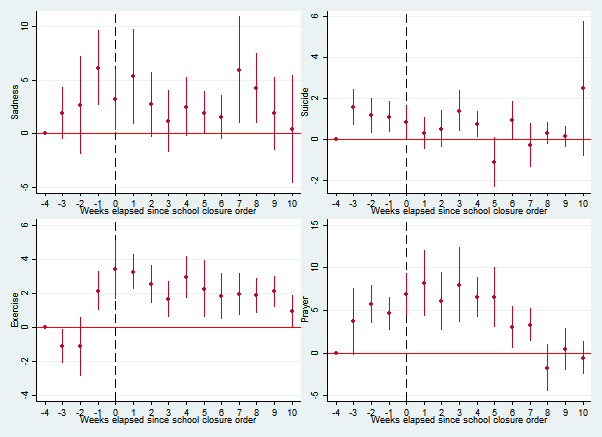


**Note:** The vertical axis shows the event-study estimates using the 2019 period as the counterfactual. The 4th week before the school closure (in 2019 or 2020) is the reference period. The models include dummies for each week from three weeks before to 10 weeks after the school closure order. In addition, controls were added for the country, year, week, and day of the week fixed effects as well as the one-day lagged number of new COVID-19 related deaths per million. Weights are applied and the standard errors which are clustered at the day level are plotted.
